# Bridging the trust gap: insights from students on rebuilding confidence in public health

**DOI:** 10.3389/fpubh.2026.1683335

**Published:** 2026-05-21

**Authors:** P. Christopher Palmedo, Lauren Rauh, Adam Doyno, Ayman El-Mohandes

**Affiliations:** Graduate School of Public Health & Health Policy, City University of New York, New York, NY, United States

**Keywords:** trust, public health, public health education, qualitative research, focus groups

## Abstract

**Objectives:**

To investigate how public health graduate students and recent alumni self-identify as builders of institutional trust within their communities.

**Methods:**

We conducted 6 focus groups of students and alumni (*n* = 37) from a public graduate school of public health in a large U.S. city in February 2024.

**Results:**

Participants articulated roles and pathways in building trust in public health and in government, and commonly find themselves in public health advocacy positions.

**Conclusion:**

Schools of public health can strengthen institutional trust through their role in training students in culturally responsive communication and related trust-building competencies.

## Introduction

Trust is a cornerstone of effective governance and indispensable to public health. When trust thrives, health programs enjoy greater acceptance and adherence and are more likely to succeed (1, 2). We distinguish between interpersonal trust (confidence in specific individuals based on personal interactions) and institutional trust (confidence in organizations and systems). Interpersonal trust, i.e., confidence in specific individuals based on personal interactions, may serve as a pathway to institutional trust (3). Both interpersonal and institutional trust drive cooperation, collaboration and creativity (4, 5). In this study, we focus on institutional trust in public health systems, while recognizing that such trust is often mediated through interpersonal relationships (e.g., family members, peers). We use the term “trust-building” to refer to both strengthening existing trust and rebuilding trust where it has eroded between communities and public health institutions. Public health interventions (e.g., masking, distancing, quarantining) rely on trust because they often require people to pay a personal cost to protect the collective good. Without trust in governments and in people, interventions can face resistance, rejection, and even disinformation campaigns against them (6). During the COVID-19 pandemic, throughout the world, low levels of trust correlated with higher infection rates (7, 8). Recent research has begun to identify specific psychological mechanisms through which trust in science can be shaped during public health crises (9).

A diverse public health workforce strengthens trust between health systems and marginalized communities (10–12). When public health professionals reflect the cultural, linguistic, and lived experiences of the populations they serve, they are better able to engage in productive communication approaches (10, 11). This alignment enhances credibility and respect, which improves institutional trust (13). Conversely, a lack of workforce diversity can contribute to lower engagement with prevention and treatment, and thus perpetuate health disparities (3, 14, 15). The research cited here strongly indicates that a trustworthy and representative workforce can lead to improved adherence to health recommendations, greater uptake of services, and better health outcomes.

Public schools of public health play an especially important role in training workforces to build trust (16). Public schools of public health enroll high proportions of students from low-income communities, especially communities of color (17, 18). Data from the Association of Schools and Programs of Public Health (ASPPH), revealed that the percentage of Black and Hispanic students enrolled in public schools averaged 29% higher than in private schools (18). Furthermore, the student population at public schools of public health is more likely to be composed of working professionals than at more expensive private schools, which have a higher proportion of younger students who recently graduated from four-year colleges (17). As a result, students from public schools are more likely to be exposed to the on-the-job experiences of health professionals than students at private schools (19). The literature confirms that increased health workforce diversity builds trust in marginalized communities (20, 21). However, less is known about how diverse members of the health workforce perceive and communicate their role as “trust-builders” within their communities and how this plays out in their work and daily lives. This study explores how graduate students at a public school of public health see their potential role as builders of trust. We also examine how students’ insights on this potential opportunity to increase community trust can be bolstered.

## Methods

We conducted six focus groups of students and recent alumni (*n* = 37) from a public graduate school of public health in a large urban city in the U.S. Most students at the school earn master’s degrees in public health (MPH). The school also offers master’s of science (MS) degrees in fields such as Health Communication and Industrial Hygiene, as well as doctoral (PhD) degrees. Focus groups were chosen as the qualitative research method because they allow for in-depth exploration of how public health students perceive their roles in their communities, facilitating rich discussions that capture varying viewpoints and experiences. We used a volunteer (self-selection) sampling approach; no purposive or stratified sampling was employed. Participants were recruited via emails sent to all students and alumni, outlining the purpose of the focus groups and offering a $50 gift card incentive to participate. All enrolled students and alumni across MPH, MS, and PhD programs were included in the email distribution. We used a web-based scheduler to set up six time slots. All focus group slots filled up within 24 h. After last-minute cancellations and other attrition, 37 students participated across the six groups. The sample reflects the gender distribution of the school’s student and alumni population (approximately 80% female).

Each focus group was sixty-minutes long and conducted over Zoom in February 2024. One team member facilitated the discussions using a semi-structured focus group guide, while another monitored attendance, provided technical support, and took notes. Sessions were recorded and transcribed verbatim. Participants shared additional perspectives through the Zoom chat, and we later integrated these comments into the transcriptions at the corresponding timestamps. The Human Research Protection Program (HRPP) at the academic institution sponsoring this study categorized this study as “quality improvement” (e.g., gaining information to better understand the student population and its contributions to trust) and provided an exemption from IRB review. In this case, the quality improvement opportunities included informing curriculum, student recruitment messaging, and institutional advancement / development.

Table 1 shows the demographic characteristics of the 37 focus group participants. At the time of the focus groups (February 2024), 41% of participants were alumni and 59% were enrolled graduate students. Recent alumni were defined as graduates within the past 0–7 years (mean: 3.1 years).

**Table 1:** Participant info/demographics total *n* = 37.

Demographic	*n*	%
Gender		
Female	31	84%
Male	6	16%
Race/ethnicity		
White	14	38%
Asian	8	22%
Black or African American	6	16%
Latino/a or Hispanic	6	16%
Two or more races	1	3%
Other	2	5%
Age range		
20–34	28	76%
35–49	7	19%
50–64	2	5%
Residence		
Within the New York Tri-State region*	30	81%
Outside of the NY Tri-State region	7	19%
Student status		
Current student	22	59%
Alumni	15	41%

*The New York Tri-State region refers to the urbanized areas around New York City, Northern New Jersey, and Western Connecticut.

### Focus group guide and questions

The focus group guide was informed by prior literature on institutional trust, health communication, and mistrust in public health systems (2, 3). Questions were designed to elicit participants’ experiences with trust, mistrust, and their perceived role in communicating health information within their communities. The focus groups opened with a discussion about the COVID-19 pandemic and the role of trust in the news media, political leadership, COVID-19 policymakers, and healthcare professionals. Participants were asked to share conversations they may have had with family, friends, and colleagues during the pandemic. The focus group guide included open-ended questions and prompts about interpersonal and institutional trust, societal developments threatening trust, how trust is built and rebuilt, etc. Participants discussed their roles as public health students and professionals in strengthening trust in science. Participants were asked to reflect on the roles and responsibilities played by their school of public health in bolstering and rebuilding trust in health programming and policymaking.

## Data analysis

The coding team consisted of a faculty member (CP) and an MPH graduate (LR), both with public health training. The researchers’ insider status created both advantages (understanding of concepts) and potential confirmation bias. Coding disagreements were resolved with attention to ensuring codes reflected participants’ language rather than our assumptions.

We conducted an inductive thematic analysis informed by grounded theory principles. Transcripts were coded iteratively using open coding, followed by grouping codes into broader thematic categories through team discussion. The two primary authors met multiple times to refine the codebook and resolve discrepancies. We used Dedoose to examine code co-occurrence and support theme development. Final themes were agreed upon through consensus. These themes were ordered to construct a logical narrative around the promise that public graduate schools of public health have for trust building, civic engagement, and increasing social capital.

After we reached consensus on final themes, we also ran the transcripts through the Chat GPT 4.0 artificial intelligence (AI) tool and asked for key themes for reference. The themes produced by the AI confirmed the inductive coding and thematic analysis conducted by the researchers and offered no significant insights or thematic directions that were not identified by the authors. The role played by this use of AI was to help identify potential “blind spots” or biases that may have been at play in the researchers’ methodology.

## Results

Through these discussions, participants reflected on the strengths and challenges they encounter as public health students and professionals. Their perspectives reveal the powerful role that public universities play in shaping a workforce capable of building trust and advancing health equity. Students at large public universities contribute diverse perspectives and strong connections to the communities they serve. Many of the students interviewed come from racially and ethnically diverse, immigrant, and/or lower-income communities, which are often disproportionately impacted by poor health outcomes. As these students advance in their public health graduate school training, they develop skills to serve as health communicators within their families, workplaces, and neighborhoods. Their perspectives contribute insights into the barriers communities face in accessing accurate health information that is culturally relevant, linguistically appropriate, and easy to understand. The following themes illustrate their experiences, concerns, and point toward solutions they see for improving public health communication and practice (see Table 2).

**Table 2:** Themes and select quotes.

Theme	Sub/Expanded themes	Select quotes
1. Diversity of public university students in a school of public health is a strength	School diversity is seen as a strength for incoming students as they appreciate the wide range of perspectives from peers. Diversity is a strength of the school, connecting it more closely with diverse communities.	*We represent such a diverse set of people that end up being on front lines.* *The City Health Department staff was happy when they could work with people from (our school) because we are representative of the community.* *The diversity of the people here has been a huge factor.* *There is an on-the-ground, applied lens here.* *My capstone about the epidemiology workforce showed this school is producing the most diverse graduates in the city.*
2. Misinformation/poor health literacy can be frustrating for those with public health training.	Participants expressed frustration and concern over the gap between their science-based education and the beliefs of family and friends. Participants recognized that their training is important to improving health and achieving health equity.	*To witness the level of misinformation being spread, and influencing our society, was just so deeply concerning.* *Coming from my (immigrant) background, I had lots of friends and family getting information from “Whatsapp University,” and different forms of video text messages. A lot of times it’s false.* *You need this kidney, but you are not moving forward with the surgery because you do not want a vaccine?* *We’re fighting misinformation from our own politicians.*
3. Public health and medicine can be elitist and condescending	Despite gaps in health literacy, practitioners must not disrespect those who question public health policy and practice Diverse cultural backgrounds often seem at odds with contemporary guidance Dismissing the inquiry only deepens the trust divide.	*In the healthcare system, we are this set of elites; only we know this information.* *The way my hospital approached COVID wasn’t the most welcoming. It was like, here’s a pamphlet, here’s information, and there you go.* *In my community, during COVID lots of herbal recipes were going around and the experts would say, Oh, no, that’s misinformation.*
4. Diverse public health students trained at a public institution are a solution to declining trust	These students and practitioners are a bridge between evidence-based practice and distrust Cultural ties make these community members more reliable messengers	*The majority of our hospital workers are Spanish speaking and during the pandemic, there was a lot of fear. I worked with the CEO to offer Spanish interpretation and we made a big impact on our standing within the community.* *People would ask me if I was OK from the vaccine. I was like, yeah, I was fine, like, I was a little sick after but nothing crazy.* *When I speak Spanish, they are like, “Oh, my God, my people!” And they feel more comfortable and trust the process.* *I’m the only Black person on my research team. About 85% of our participants are of African descent or are our Hispanic brothers and sisters. We do not see a lot of researchers of color working with those communities.* *I see myself as that bridge. We’re community health workers. I’m having to represent public health, the Department of Health, my life, and then share this information with my family and be a part of rebuilding that trust. I understand where they are coming from, and it makes perfect sense.* *Telling my Chinese immigrant family what I know about vaccines helped bridge the gap because they trust me. I offer a professional transfer of information. I’m the daughter of restaurant owners.*

### Theme 1: The diversity of public university students is an important strength

Student diversity at a graduate school of public health is a strength for the students, for the school, and for the communities served by those students. Participants told us that public health graduate students and alumni who come from racially and ethnically diverse, immigrant, and lower-income backgrounds often remain connected to these communities while pursuing their education. They apply public health principles in real-world settings and share health information with their families, neighbors, and professional networks. According to the participants, their lived experience and their education together prepare them to address the barriers faced by communities in need of improved health outcomes (e.g., “We’re representative of the community”).

### Theme 2: Addressing misinformation and poor health literacy can be frustrating for students

Participants described the frustration of confronting misinformation in their daily lives (e.g., “…the level of misinformation being spread, and influencing our society, was just so deeply concerning”). Some recounted extreme cases, such as patients refusing surgery because of a vaccine requirement. Others spoke about challenges within their own immigrant communities, where language barriers and reliance on informal networks made misinformation harder to address. They spoke about how public health professionals were unprepared to counter these dynamics, particularly in communities already skeptical of institutions. The discussion stressed that misinformation is not necessarily an individual failing but something widely shaped by broader social contexts. Participants said that when information is not tailored to a community’s language, literacy levels, or cultural context, it can create space for misinformation that feels more familiar or trustworthy to the recipient.

### Theme 3: Public health and medicine can be elitist and condescending

Participants described a public health and medical system where messaging can be tone-deaf, detached, and impersonal (e.g., “The way my hospital approached COVID wasn’t the most welcoming”). Some contended that public health guidance is often delivered as a set of instructions rather than a conversation, leaving little room for questions, concerns, or the realities of people’s lives. During COVID-19, many saw this play out firsthand—hospitals handed out pamphlets instead of having real discussions, and public health leaders dismissed traditional remedies in ways that felt condescending rather than informative. Participants understood the need to combat misinformation, but they also recognized that skepticism toward authority is deeply ingrained, especially in communities with histories of government neglect or abuse. People are not simply misinformed—they are cautious, protective, and often acting in the best interest of their families. Dismissing that instinct as ignorance only deepens the divide. If public health professionals want trust, they must offer the same respect they expect in return.

Participants also noted that while research and data are widely available, they are often misinterpreted by the public or deliberately distorted by those spreading disinformation. This creates a landscape where multiple viewpoints—including inaccurate ones—can present themselves as evidence-based. Public health officials and medical personnel often respond by leaning on their training and expertise. However, as participants noted, this dynamic can alienate people who are skeptical or simply seeking reassurance about public health guidance.

The COVID-19 pandemic made these tensions more visible; messages were often rigid directives without acknowledging fears or historical reasons for distrust. Participants expressed disappointment that the public health system was quick to reject alternative health practices in their communities rather than meaningfully engaging with people. Distrust of medical and elite institutions is not new, and they argued that public health should face this phenomenon head-on.

### Theme 4: Diverse students trained in public health at a public institution are a solution to declining trust

Participants saw themselves as exceptionally positioned to bridge the trust gap between health institutions and the communities they serve (e.g., “I see myself as that bridge. We’re community health workers”). They carried lived experience and formal training, allowing them to communicate health information with language that resonates with their families, neighbors, and friends. Representation was discussed, particularly in communities where language barriers and historical distrust of institutions shaped attitudes toward healthcare. Spanish-speaking students, for example, discussed how being able to engage in someone’s native language could shift an interaction from skepticism to trust. Others reflected on how their cultural ties made them reliable messengers, whether answering vaccine concerns at registration sites or countering incorrect information in casual conversations. The ability to connect personally, not as distant experts, but as familiar and trusted figures, is a critical asset that public health and government institutions should not overlook.

## Discussion

This study examined how public health graduate students and recent alumni perceive their role as builders of institutional trust. Findings highlight the important role public health graduate students can play in bridging the trust gap between institutions and local communities. Beyond the specific themes identified, several cross-cutting insights emerge. Participants consistently described public health education as embodying trustworthiness through humility, cultural awareness, and civic responsibility. This reflects similar findings by Lansing et al. (22), who identified important trust-building elements such as collaboration, transparency, and equity, all of which are explored in public health curricula (23). Graduate-trained public health professionals complement the role of community health workers, whose trust-building occurs primarily through direct service. Public health students’ trust-building capacity emerges from academic institutions that are strictly committed to exploring science and scholarship. The dual perspective of participants (science-trained and community-based) positions them as empathetic translators of evidence. Schools of public health cultivate professionals who can navigate the political complexities of rebuilding community confidence in public institutions.

These findings have implications for how schools of public health can intentionally cultivate and deploy students’ trust-building capacity. Public health education can formalize specific practices and competencies into its curricula. For example, schools can integrate explicit training in recognizing and navigating institutional distrust, and include historical contexts (Henrietta Lacks case, Baltimore lead studies, etc.) that have shaped community skepticism. Case-based courses can include opportunities to explore scenarios where elitism or condescension play a role in damaging trust between institutions and communities. Communications courses should include health literacy, media training, and public speaking to prepare students to engage with policymakers, media, and community leaders. Schools are also logical conveners for forums and discussions to explore different perspectives and viewpoints. Explicitly incorporating trust-building into Council on Education for Public Health (CEPH) competency frameworks would signal its importance and encourage schools to integrate it into their curricula. To operationalize these findings, philanthropic organizations can fund scholarships for public health students or bridge programs that help direct diverse graduates to community-facing roles, such community health worker residencies, fellowships in health departments, or peer mentorships.

Lessons can be drawn from established programs that have successfully connected learning, service, and credibility. The CDC’s Public Health Associate Program demonstrates how early-career professionals can strengthen local trust through long-term community placements. The DeBeaumont Foundation’s PH WINS framework collects data on the demographics of the workforce and captures individual public health workers’ perspectives on key issues such as workforce engagement and morale, training needs, and emerging concepts in public health. Other existing trust-building mechanisms within academic public health infrastructure include the community health worker / promotores model, where trusted messengers share language, culture, and lived experience with the residents they serve. Health agencies, public universities, and government funders must invest in these kinds of mechanisms through stable funding, durable institutional commitments, and clear professional pathways. Inconsistent or short-term support weakens capacity to build and sustain public trust (24).

These recommendations face implementation challenges. Recent policy changes, budget constraints, and political pressures on public health may complicate efforts to expand and support diverse public health education (25). Budget cuts to public health programs, attacks on the health system from the Executive branch of government, financial pressures that push students away from community-oriented roles, and the increased scale of misinformation campaigns using artificial intelligence continue to impose sustained pressure on the trust that society places on public health institutions and policies (26).

## Limitations

This qualitative study explored students’ views of their position as trust-builders, recognizing that interpersonal trust in individual students does not automatically translate to institutional trust in public health systems. Because data were drawn from a single public graduate school, results reflect the context of the institution, and do not capture the full range of attitudes found in rural settings, outside the U.S., etc. The study sought depth of thought over representativeness, and students and alumni most engaged with the school may have been more likely to participate. Furthermore questions foregrounded COVID-19 and trust in advance, which may have primed participants toward particular narratives. As a result, the experience may reflect more interest and passion for the topic than that which exists in the wider body of students and alumni. Our analysis reflects a normative orientation shared by participants and researchers, framing students as civic actors positioned to repair trust.

## Conclusion

Public health graduate students, especially those at public institutions, are future leaders already engaged in rebuilding trust, often in ways that traditional health institutions have failed to fully achieve (27, 28). Future research involving interviews with leaders from underrepresented communities could add valuable nuance to the inquiry explored in this study. Further studies should investigate the support systems and opportunities (e.g., basic needs support, leadership training) for students like these who look ahead to health and community-based leadership positions.

Trust is foundational to building strong public institutions and healthy societies. Funding sources, from government to philanthropy, should invest in people and institutions that contribute to rebuilding trust. This study has shown that an established public school serving a diverse student body is a powerful engine to rebuilding trust in public health, and one that deserves strong consideration for public and private investment.

## Data Availability

The original contributions presented in the study are included in the article/supplementary material, further inquiries can be directed to the corresponding author.

## References

[1] Gilson L. Trust and the development of health care as a social institution. Soc Sci Med. (2003) 56:1453–68. doi: 10.1016/s0277-9536(02)00142-9, 12614697

[2] Ozawa S Stack ML. Public trust and vaccine acceptance-international perspectives. Hum Vaccin Immunother. (2013) 9:1774–8. doi: 10.4161/hv.24961, 23733039 PMC3906280

[3] Richmond J Anderson A Cunningham-Erves J Ozawa S Wilkins CH. Conceptualizing and measuring trust, mistrust, and distrust: implications for advancing health equity and building trustworthiness. Annu Rev Public Health. (2024) 45:465–84. doi: 10.1146/annurev-publhealth-061022-044737, 38100649 PMC11156570

[4] Schiffman L Thelen ST Sherman E. Interpersonal and political trust: modeling levels of citizens’ trust. Eur J Mark. (2010) 44:369–81. doi: 10.1108/03090561011020471

[5] Kaasa A Andriani L. Determinants of institutional trust: the role of cultural context. J Inst Econ. (2022) 18:1–21. doi: 10.1017/S1744137421000199

[6] Kisa S Kisa A. A comprehensive analysis of COVID-19 misinformation, public health impacts, and communication strategies: scoping review. J Med Internet Res. (2024) 26:e56931. doi: 10.2196/56931, 39167790 PMC11375383

[7] Salvador CE Berg MK Yu Q San Martin A Kitayama S. Relational mobility predicts faster spread of COVID-19: a 39-country study. Psychol Sci. (2020) 31:1236–44. doi: 10.1177/0956797620958118, 32915703

[8] Bollyky TJ Castro E Aravkin AY Bhangdia K Dalos J Hulland EN . Assessing COVID-19 pandemic policies and behaviours and their economic and educational trade-offs across US states from Jan 1, 2020, to July 31, 2022: an observational analysis. Lancet. (2023) 401:1341–60. doi: 10.1016/S0140-6736(23)00461-0, 36966780 PMC10036128

[9] Borinca I Griffin SM McMahon G Maher P Muldoon OT. Nudging (dis)trust in science: exploring the interplay of social norms and scientific trust during public health crises. J Appl Soc Pyschol. (2024) 54:487–504. doi: 10.1111/jasp.13053

[10] Cohen JJ Gabriel BA Terrell C. The case for diversity in the health care workforce. Health Aff. (2002) 21:90–102. doi: 10.1377/hlthaff.21.5.90, 12224912

[11] Coronado F Beck AJ Shah G Young JL Sellers K Leider JP. Understanding the dynamics of diversity in the public health workforce. J Public Health Manag Pract. (2020) 26:389–92. doi: 10.1097/PHH.0000000000001075, 31688743 PMC7190406

[12] Hunt DV Layton D Prince S (2015) Why diversity matters. Available online at: https://www.mckinsey.com/capabilities/people-and-organizational-performance/our-insights/why-diversity-matters (Accessed April 18, 2024)

[13] Bishu SG Kennedy AR. Trends and gaps: a meta-review of representative bureaucracy. Rev Public Personnel Adm. (2020) 40:559–88. doi: 10.1177/0734371X19830154

[14] Carlisle BL Murray CB. "The role of cultural mistrust in health disparities". In: The Wiley Encyclopedia of Health Psychology. Hoboken, NJ: John Wiley & Sons, Ltd (2020)

[15] Corbie-Smith G Thomas SB St George DMM. Distrust, race, and research. Arch Intern Med. (2002) 162:2458–63. doi: 10.1001/archinte.162.21.2458, 12437405

[16] Gomez LE Bernet P. Diversity improves performance and outcomes. J Natl Med Assoc. (2019) 111:383–92. doi: 10.1016/j.jnma.2019.01.006, 30765101

[17] Oyo A. Workforce Diversity and Health Equity: The Role of Schools of Public Health. New York, NY: CUNY Academic Works (2022).

[18] Freudenberg N Klitzman S Diamond C El-Mohandes A. Keeping the “public” in schools of public health. Am J Public Health. (2015) 105:S119–24. doi: 10.2105/AJPH.2014.302534, 25706006 PMC4339992

[19] Resnick B Leider JP Riegelman R. The landscape of US undergraduate public health education. Public Health Rep. (2018) 133:619–28. doi: 10.1177/0033354918784911, 30084738 PMC6134569

[20] Alsan M Garrick O Graziani G. Does diversity matter for health? Experimental evidence from Oakland. Am Econ Rev. (2019) 109:4071–111. doi: 10.1257/aer.20181446

[21] LaVeist TA Pierre G. Integrating the 3Ds—social determinants, health disparities, and health-care workforce diversity. Public Health Rep. (2014) 129:9–14. doi: 10.1177/00333549141291s204, 24385659 PMC3863706

[22] Lansing AE Romero NJ Siantz E Silva V Center K Casteel D . Building trust: leadership reflections on community empowerment and engagement in a large urban initiative. BMC Public Health. (2023) 23:1252. doi: 10.1186/s12889-023-15860-z, 37380973 PMC10304359

[23] Hobson KA Coryn CLS Fierro LA Sherwood-Laughlin CM. Instruction of evaluation competencies in council on education for public health (CEPH)-accredited master of public health (MPH) degree programs. Am J Eval. (2019) 40:590–606. doi: 10.1177/1098214019845510

[24] Sheehan BE. Maintaining trust in uncertain times: funding pauses and the ethical cost to community-engaged research. American J of Comm Psychol. (2025) 77:291–4. doi: 10.1002/ajcp.70028, 41236174 PMC13007744

[25] Association of Schools and Programs of Public Health. Department of Education Proposal Excludes Public Health Degrees from “Professional Degree” Definition. Washington, DC: Association of Schools and Programs of Public Health (2025).

[26] Saeidnia HR Hosseini E Lund B Tehrani MA Zaker S Molaei S. Artificial intelligence in the battle against disinformation and misinformation: a systematic review of challenges and approaches. Knowl Inf Syst. (2025) 67:3139–58. doi: 10.1007/s10115-024-02337-7

[27] Best AL Fletcher FE Kadono M Warren RC. Institutional distrust among African Americans and building trustworthiness in the COVID-19 response: implications for ethical public health practice. J Health Care Poor Underserved. (2021) 32:90–8. doi: 10.1353/hpu.2021.0010, 33678683 PMC7988507

[28] Bogart LM Ojikutu BO Tyagi K Klein DJ Mutchler MG Dong L . COVID-19 related medical mistrust, health impacts, and potential vaccine hesitancy among black Americans living with HIV. J Acquir Immune Defic Syndr. (2021) 86:200–7. doi: 10.1097/QAI.0000000000002570, 33196555 PMC7808278

